# SLCO1B1*5 is protective against non-senile cataracts in cohort prescribed statins: analysis in a British-South Asian cohort

**DOI:** 10.1038/s41397-023-00307-w

**Published:** 2023-05-23

**Authors:** Emma F. Magavern, David A. van Heel, Genes & Health Research Team, Damian Smedley, Mark J. Caulfield

**Affiliations:** 1 William Harvey Research Institute, Queen Mary University of London, London, EC1M 6BQ UK; 2 Blizard Institute, Queen Mary University of London, London, E1 2AB UK

**Keywords:** Therapeutics, Dyslipidaemias

## Abstract

**Background:**

Reported association between statin use and cataract risk is controversial. The *SLCO1B1* gene encodes a transport protein responsible for statin clearance. The aim of this study was to investigate a possible association between the SLCO1B1*5 reduced function variant and cataract risk in statin users of South Asian ethnicity.

**Methods:**

The Genes & Health cohort consists of British-Bangladeshi and British-Pakistani participants from East London, Manchester and Bradford, UK. SLCO1B1*5 genotype was assessed with the Illumina GSAMD-24v3-0-EA chip. Medication data from primary care health record linkage was used to compare those who had regularly used statins compared to those who had not. Multivariable logistic regression was used to test for association between statin use and cataracts, adjusting for population characteristics and potential confounders in 36,513 participants. Multivariable logistic regression was used to test association between SLCO1B1*5 heterozygotes or homozygotes and cataracts, in subgroups having been regularly prescribed statins versus not.

**Results:**

Statins were prescribed to 35% (12,704) of participants (average age 41 years old, 45% male). Non-senile cataract was diagnosed in 5% (1686) of participants. An apparent association between statins and non-senile cataract (12% in statin users and 0.8% in non-statin users) was negated by inclusion of confounders. In those prescribed a statin, presence of the SLCO1B1*5 genotype was independently associated with a decreased risk of non-senile cataract (OR 0.7 (CI 0.5–0.9, p 0.007)).

**Conclusions:**

Our findings suggest that there is no independent association between statin use and non-senile cataract risk after adjusting for confounders. Among statin users, the SLCO1B1*5 genotype is associated with a 30% risk reduction of non-senile cataracts. Stratification of on-drug cohorts by validated pharmacogenomic variants is a useful tool to support or repudiate adverse drug events in observational cohorts.

## Clinical perspective

### What is new?

- Statin use is not independently associated with an increased risk of non-senile cataracts.
- Presence of the SLCO1B1*5 genetic variant leading to increased exposure to statins is associated with a decreased risk of non-senile cataracts.
- Cross-sectional analysis of a cohort linking medication use and validated pharmacogenes with purported adverse drug reaction can support pharmacovigilance.

## What are the clinical implications?

- This study supports cardiovascular prescribers in counseling patients that statins are not independently associated with increased risk of non-senile cataracts and that in those with a SLCO1B1*5 allele, use of statins is associated with decreased risk of a non-senile cataract.
- Low-income settings without ready access to cataract surgery and with high prevalence of cardiometabolic disease may prevent some non-senile cataracts by statin use in those with SLCO1B1*5 allele.
- Addressing concerns about statin links with cataracts may enhance medication compliance in use of statins for primary and secondary cardiovascular prevention.

## Introduction

Statins are Hydroxymethylglutaryl-Coenzyme A (HMG-CoA) reductase inhibitors indicated in the treatment of primary and secondary prevention for cardiovascular disease as well as dyslipidemia [1]. They are among the most prescribed medications, second only to proton pump inhibitors in a study of English prescribing patterns [2]. A large USA based study showed that in 2013, 27.8% of adults over the age of 40 were prescribed a statin [3]. As a result of this widespread use, adverse drug reactions associated with statins have attracted significant attention [4].

Cataracts are a leading cause of blindness world-wide, particularly problematic in low-income and middle-income countries with less access to surgical interventions [5]. The reported association between statin use and cataract risk is controversial and bi-directional [4]. While some large observational studies, randomized control trials, and meta-analyses have found statins to have a protective effect on cataracts, others have found an association with increased risk of cataracts, and many studies have found no significant association in either direction [4, 6–10]. Though a systematic review and meta-analysis of observational studies suggested a small increase in cataracts associated with statin use (OR: 1.11 (95% CI: 1.02–1.21); *P* = 0.017), results were heterogeneous and likely impacted by residual confounding [11].

Observational studies can be confounded by the presence of cardio-metabolic risk factors for cataracts which are also indications for statins, and randomized controlled trials include selective populations and don’t control for population level genetic differences. The sole study to use genetics as a tool to assess the relationship between statins and cataracts mimicked the LDL lowering effect of statins in isolation. As statins are known to have diverse mechanisms of action, including decreased inflammatory proprieties independent of LDL impact, this approach will model only one aspect of statin association with cataracts [12, 13].

The solute carrier organic anion transporter family member 1B1 (*SLCO1B1)* gene encodes the transporter protein OATP1B1 [14]. OATP1B1 is responsible for the active intrahepatic transport, and subsequent clearance, of statins [14]. SLCO1B1*5 is a polymorphism associated with increased exposure to statins as shown in pharmacokinetic studies, and increased risk of statin related adverse drug events, such as myopathy and myalgia [14–16]. SLCO1B1*5 prevalence varies substantially between different ethnic groups. The literature reports SLCO1B1*5 as present in 1% of African populations, 4% of South Asians, 12% of East Asians, 13% of Americans, and 16% of Europeans [17, 18]. South Asian ancestry populations suffer from a particularly high prevalence of cardiometabolic disease, therefore exploring statin related adverse drug reactions in this population is important [19].

Association between SLCO1B1*5 and cataract risk in statin users has not been characterized, and stratification by SLCO1B1*5 genotype in statin users and non-users offers a valuable approach to clarifying the relationship between statin use and cataracts.

The aim of our study was to use a genetic proxy for increased statin exposure by presence of the SLCO1B1*5 allele in a large cohort of more than 36 thousand participants to elucidate the relationship between statins and cataracts.

## Methods

### The Genes & Health cohort

The Genes & Health cohort consists of British-Bangladeshi and British-Pakistani participants from East London, Manchester and Bradford [20]. The cohort has been broadly characterized in a prior publication [20].The Genes & Health (G&H) cohort data was accessed with approval of the study executive committee. The G&H study has obtained ethical approval, 14/LO/1240, from London South East NRES Committee of the Health Research Authority, dated 16 September 2014. Volunteers provided DNA via a saliva sample and consented to link study data with electronic health records [20]. As described in prior publications, participants were genotyped using the Illumina GSAMD-24v3-0-EA chip [21]. Human build 38 of the genome research consortium was used for this work.

### Characterization of SLCO1B1 genotype in the G&H cohort

The SLCO1B1*5 genotype was extracted from the data set using PLINK 2.0 [22, 23]. The *5 allele was defined as c.521T>C, rs4149056 (chr12:21178615 (GRCh38)). The population was in Hardy Weinberg equilibrium (HWE) for this single nucleotide polymorphisms (SNP) and there was no substantial missingness (Supplementary Table 1). The minor allele frequency (MAF) of the allele was 0.04 (Supplementary Table 1). Subsequent analysis was done in Rstudio [24].

### Medication data from primary care

**Fig. 1:**
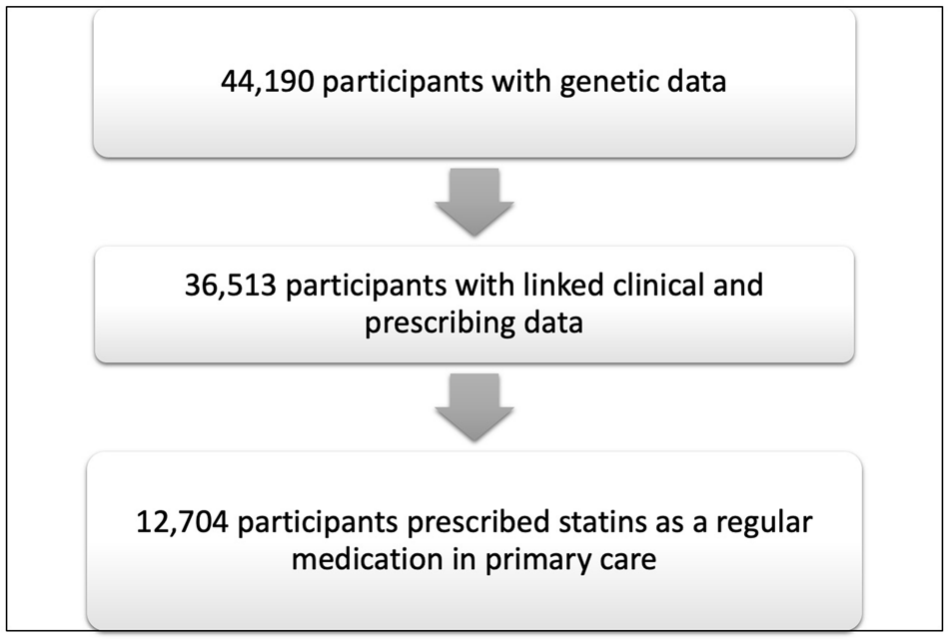
Study cohort overview.

**Fig. 2:**
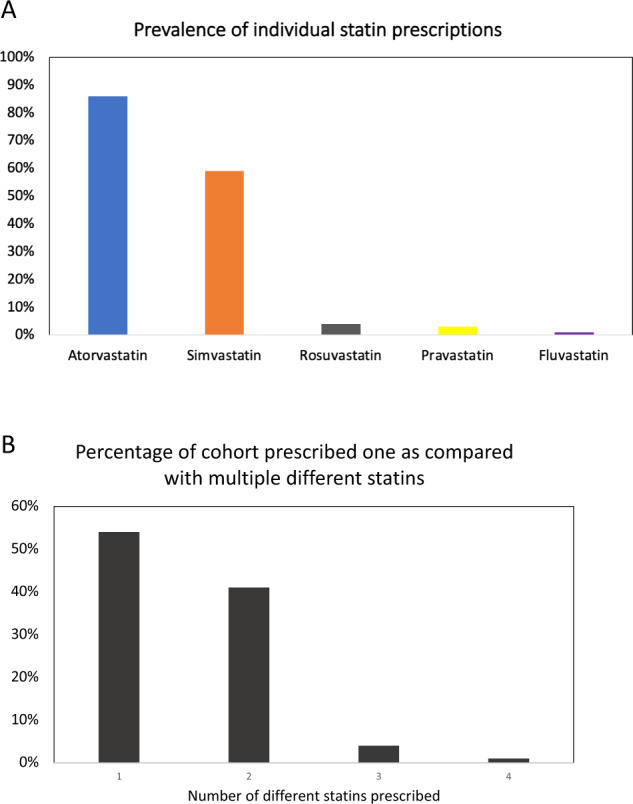
Statin prescribing prevalence and prevalence of exposure to multiple different statins. **A** Prevalance of exposure to specific statin agents within the cohort prescribed statins, in descending order of prevalence. **B** Prevalence of multiple different statin agent exposures in primary care. In the cohort prescribed statins (N 12704) it was common to have been prescribed 2 different agents within the class of statins. The odds of having been prescribed more than 1 different statin did not differ in presence or absence of SLCO1B1*5 allele using fisher exact test (OR 0.9 CI 0.8–1.0, *p* 0.14).

Medication use was assessed from linkage with primary care via participating clinical commissioning groups (CCGs) including Barking, Havering and Redbridge (BHR), Tower Hamlets (TH), City and Hackney, Waltham Forest (WF) and Newham (N). Our study population was constituted by *N* = 36,513 individuals who had genetic and clinical data, including medications (Fig. 1). Participants were assigned to the ever-used statins group if they had any record of a statin on the ordinary prescription list (versus short term prescriptions) from primary care. Participants who did not meet this criterion were assigned to the never-used statin sub-group. Type of statins used included all those available in the UK: atorvastatin, simvastatin, rosuvastatin, pravastatin, fluvastatin (Fig. 2A). Pharmacokinetic data shows that there is agent specific variation in the increase in area under the curve associated with the SLCO1B1*5 allele and statin exposure [16, 25]. The effect is most pronounced for simvastatin and atorvastatin [26]. Pooling statins as a class exposure was chosen to optimize power but may also bias against or under-estimate agent specific signal detection.

### G&H curated phenotypes

G&H curated phenotypes were used in this analysis. These were generated using ICD10 codes, SNOMED codes, and Office of Population Censuses and Surveys (OPCS) codes from linkage with electronic health records, including Barts Health, NHS digital, Bradford teaching hospitals and primary care clinical commissioning groups (CCGs). The code used to generate the curated phenotypes is openly available in GitHub and signposted on the G&H website [27]. In summary, UK Biobank methodology for identifying first occurrence of diagnoses was followed with the addition of primary and secondary care SNOMED code data [27, 28]. Non-senile cataracts were defined by ICD10 code H26. Senile cataracts were defined by ICD10 code H25. Diabetes (DM) included E10; type 1 diabetes mellitus, E11; type 2 diabetes mellitus, E13; other specified diabetes mellitus, E14; unspecified diabetes mellitus. Dyslipidemia was defined by ICD 10 code E78. Obesity was defined by ICD10 code E66. Chronic Kidney Disease was defined by ICD10 code N18. Hypertension was defined by ICD 10 code I10. Ischemic heart disease (IHD) was defined by ICD 10 codes I21; acute myocardial infarction, I24; other acute ischemic heart diseases, and I25; chronic ischemic heart disease. Peripheral vascular disease (PVD) was defined by ICD10 code I73.

### G&H curated principal components

G&H has curated principal components as referenced in a prior publication [21]. The first two of these were used to control for population stratification in our analysis.

### Statistical methods

Multivariable logistic regression was used to test for association between statin use and cataracts, adjusting for population characteristics and potential confounders by inclusion of the listed cardio-metabolic conditions and characteristics as variables. Multivariable logistic regression was used to test association between SLCO1B1*5 containing diplotypes and cataracts, adjusting for age at recruitment, gender, cardiometabolic risk factors, and two principal components in sub-groups having ever or never been prescribed statins. Fisher’s exact test was used to compare cohort characteristics (Table 1 and Supplementary Table 2).

**Table 1:** Cohort demographics, genotype, and disease prevalence.

Medication use	All participants (*N* = 36,513)	Prescribed Statin (*N* = 12,704)	Not Prescribed Statin (*N* = 23,809)	*P* value
Average age at enrollment (SD)	41 years old (±14 years)	53 years old (±12 years)	34 years old (±10 years)	<2.2 e−16
Male % (*n*)	45 (16,465)	58 (7395)	38 (9070)	<2.2 e−16
Obesity % (*n*)	17 (6140)	23 (2928)	13 (3212)	<2.2 e−16
Diabetes % (*n*)	16 (6024)	42 (5377)	3 (647)	<2.2 e−16
Hypertension % (*n*)	19 (6801)	46 (5861)	4 (940)	<2.2 e−16
Dyslipidemia % (*n*)	21 (7576)	54 (6831)	3 (745)	<2.2 e−16
CKD % (*n*)	5.9 (2155)	15 (1935)	0.9 (220)	<2.2 e−16
PVD % (*n*)	1.3 (477)	2 (274)	0.9 (203)	<2.2 e−16
IHD % (*n*)	7.5 (2741)	21 (2629)	0.5 (112)	<2.2 e−16
Cataracts (all) % (*n*)	5.4 (1973)	14 (1764)	0.9 (209)	<2.2 e−16
Cataracts, non-senile % (*n*)	4.6 (1686)	12 (1507)	0.8 (179)	<2.2 e−16
SLCO1B1*5 homozygote or heterozygote % (*n*)	8.6 (3122)	8.8 (1115)	8.4 (2007)	0.3

*CKD* chronic kidney disease, *IHD* ischemic heart disease, *PVD* peripheral vascular disease.

## Results

The average age at enrollment was 41 years old (±14 years) and 45% of participants were males. The cohort was characterized by a high prevalence of cardio-metabolic conditions including obesity (17%), diabetes (16%), hypertension (19%), and dyslipidemia (21%) (Table 1). 35% of G&H participants with linked medication data **(**12,704/36,513) had been prescribed a statin as an ordinary medication in primary care (Table 1, Fig. 1). Figure 2A illustrates the prevalence of individual medications within the statin class. Atorvastatin and simvastatin were the most commonly prescribed agents (Fig. 2A). Fluvastatin was not commonly prescribed. Some participants had been prescribed multiple different statins (Fig. 2B). 54% had only been prescribed 1 agents, while 41% had been prescribed 2 different statins and 4% had been prescribed 3 different types of statin. It was rare to have been prescribed 4 different types of statins (Fig. 2B).

1686 Participants (5%) had a non-senile cataract. 995 Participants (3%) had a senile cataract. 668 participants were diagnosed with both senile and non-senile cataracts. When stratified by statin use, 12% of participants who had been prescribed statins had a diagnosis of non-senile cataract, compared with 0.8% of those not prescribed a statin (Supplementary Table 2). The association between statin use and non-senile cataracts was not independent after controlling for confounding conditions associated with both CV and cataract risk, and population stratification (Table 2).

**Table 2:** Association of statin use with non-senile cataracts adjusted for confounders: dyslipidemia, obesity, hypertension, CKD, PVD, diabetes, IHD, age at recruitment, sex, and two principal components.

	OR	CI	*P* value
**Statin use**	**1.0**	**0.8–1.2**	**0.97**
Dyslipidaemia	1.7	1.4–1.9	2.6e−10
Obesity	1.2	1.1–1.4	0.005
Hypertension	2.0	1.7–2.4	2.8e−16
CKD	1.3	1.1–1.5	0.0004
PVD	1.3	0.9–1.8	0.12
Diabetes	2.0	1.7–2.3	2e−16
IHD	0.8	0.7–0.9	0.003
Age at recruitment (Years)	1.1	1.1–1.1	<2e−16
Female sex	1.1	1.0–1.3	0.06

*CKD* chronic kidney disease, *IHD* ischemic heart disease, *PVD* peripheral vascular disease.

The exposure of interest for our analysis is shown in bold.

**Fig. 3:**
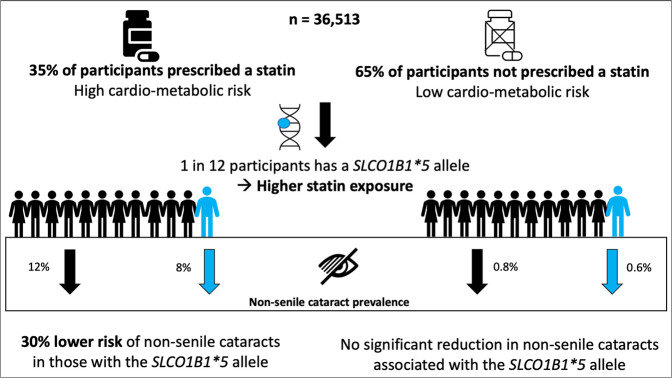
Study results overview. The SLCO1B1*5 allele was present in 8% of participants. 35% of the study population were prescribed statins. In the cohort prescribed statins, there was a 30% lower odds of non-senile cataracts diagnosis in those with a SLCO1B1*5 allele as compared with those who did not have a SLCO1B1*5 allele. There was no significant reduction in cataract risk in those with a SLCO1B1*5 not exposed to statins.

8% of the whole studied population had a SLCO1B1*5 allele (Fig. 3). Only 0.2% of the cohort were homozygous for the *5 allele. There was no significant difference in SLCO1B1*5 genotype between those prescribed only 1 statin as compared with those prescribed more than 1 different type of statin (OR 0.9 CI 0.8–1.0, *p* 0.14).

In the cohort who had been prescribed statins, 8% of those with a SLCO1B1*5 allele had a diagnosis of non-senile cataract as compared with 12% of those without a SLCO1B1*5 allele (Supplementary Table 2). The presence of the SLCO1B1*5 genotype was significantly associated with a lower risk of non-senile cataract, controlling for age at enrollment, gender, principal components, and co-morbidities (OR 0.7 CI 0.5–0.9, *p* 0.007) (Table 3). The significant association between SLCO1B1*5 genotype and non-senile cataract diagnosis was not present in the subgroup not prescribed statins (*p* 0.5). The association between SLCO1B1*5 genotype and senile cataract diagnosis was not significant (*p* 0.2). Cohort characteristics stratified by statin use and by genotype are outlined in Supplementary Table 2.

**Table 3:** Multivariable logistic regression assessing association between SLCO1B1*5 genotype presence and non-senile cataract diagnosis in the on-statin cohort.

	OR	CI	*P* value
SLCO1B1*5	**0.7**	**0.5–0.9**	**0.007**
Dyslipidaemia	1.7	1.4–2.0	2.3e−10
Obesity	1.2	1.0–1.4	0.02
Hypertension	1.9	1.6–2.2	7.1e−12
CKD	1.3	1.1–1.5	0.0006
PVD	1.2	0.8–1.6	0.3
Diabetes	2.0	1.7–2.3	<2e−16
IHD	0.8	0.7–1.0	0.01
Age at recruitment (per year)	1.1	1.1–1.1	<2e−16
Female sex	1.1	1.0–1.3	0.04

Adjusted for dyslipidaemia, obesity, hypertension, CKD, PVD, diabetes, IHD, as well as age at enrolment, gender, and two principal components.

*CKD* chronic kidney disease, *IHD* ischemic heart disease, *PVD* peripheral vascular disease.

The exposure of interest for our analysis is shown in bold.

## Discussion

Our study found an association between statin use and a high prevalence of non-senile cataracts, but this appeared to be entirely explained by the burden of cardiometabolic risk factors (which was expectedly higher in individuals who were prescribed statins). In a large cohort of British South Asian ancestry participants who were prescribed statins, individuals carrying a SLCO1B1*5 allele had a 30% lower risk of developing non-senile cataract in comparison with individuals who did not carry this polymorphism known to lead to higher systemic exposure to statins (Fig. 3). As the SLCO1B1*5 allele has been linked with muscle related ADRs, we have undertaken this two-step analysis to show that the SLCO1B1*5-statin association with decreased cataract prevalence is not likely to be due to decreased exposure to statin through lower doses or non-compliance in those with SLCO1B1*5. If the protective SNP-drug effect seen were due to less statin exposure in those with a SLCO1B1*5 allele we would expect to see an association of statin use with cataracts independent of confounding factors in the first step of the analysis. We show that such an association is not present.

Our findings add a significant piece of the puzzle in the controversy regarding association between statins and cataracts, suggesting that pharmacogene association with statins are responsible for decreased non-senile cataract risk by higher exposure to statins. This is proof of concept that stratification by pharmacogene in observational on-drug cohorts can be helpful in clarifying drug association with putative adverse drug events. The G&H South Asian ancestry cohort is uniquely suited for this study due to high rates of cardiometabolic disease, and therefore a high-risk profile for non-senile cataracts.

These novel results represent the first exploration of pharmacogene-statin interaction in association with cataracts and are reassuring given the prevalence of statin prescription in the G&H community and broader population. They also account for conflicting results in the literature of bi-directional statin association with cataracts. Many prior studies have not reported on ethnic composition of the study cohort, so it is difficult to assess potential implications of our findings in interpretation of prior work. On a population level the protective effect of statins associated with SLCO1B1*5 would be amplified in European ancestry populations and minimal in African ancestry populations, due to diverse prevalence of SLCO1B1*5 in these populations, if prescription rates and co-morbidities are constant. Therefore, it seems unlikely to be accidental that the sole RCT reporting a protective effect of statins on cataracts included a 99.7% Caucasian cohort [9, 29]. It also seems quite likely that pooling studies from diverse populations without controlling for ancestry may yield conflicting results, particularly if both disease prevalence and allele prevalence vary across populations. None of the prior studies have included pharmacogenomic data.

Though the SLCO1B1*5 genotype was protective in association with non-senile cataracts there was no significant association with senile cataracts. This may be simply because the numbers of participants with senile cataracts were smaller and therefore this study was underpowered to find an association, if present, or may be because the pathophysiology of senile versus non-senile cataracts is different.

### Clinical implications

This study suggests that individuals with a SLCO1B1*5 allele who are prescribed statins are at lower risk of developing a non-senile cataract. Such individuals at high risk of non-senile cataract from cardiometabolic conditions may reduce this risk by a third if they take a statin. It thus highlights potential therapeutic opportunities in cataract prevention. It also underlines potential to use observational cohort data in conjunction with pharmacogene information to elucidate purported adverse drug reactions, an approach which had not been applied prior to this question.

Compliance to medications may be variable and dependant on numerous factors, including strength of counselling and depth of information available to patients. Certainly, knowledge of pharmacogenetic background and of dramatic reduction in risk of developing a potentially disabling condition may have a significant impact on patients’ attitude toward statins, and thereby compliance. There is international consensus that pharmacogenomic testing is entering mainstream cardiovascular medicine, and therefore patients may well know if they have a SLCO1B1*5 allele in the near future [26].

### Limitations

This study was not equipped to differentiate the association between individual statins and non-senile cataracts, as opposed to class effect. This was due to limited number of non-senile cataract events and unequal prescription of individual statins to individuals in the cohort. Likewise, due to unequal distribution of different statin agents and dosages as well as lack of timeline data this study was not equipped to assess effects of different dosages.

The results presented here have pooled participants who are homozygous for the SLCO1B1*5 allele and those who are heterozygous. This was because of limited number of participants homozygous for the SLCO1B1*5 allele in this population (only 26 participants with linked clinical and medication data were homozygous for SLCO1B1*5 and had been prescribed a statin).

The dates of events were not available. Thus, we are unable to link time of statin use and time of cataract. We did not quantify time on statin prior to cataract for the same reason (due to lack of timeline data). However, the presence of the statin medication as a regular rather than short term medication assumes chronic use.

Despite these limitations, the relationship between the SLCO1B1*5 genotype and reduced non-senile cataract risk only existed in the cohort who had been prescribed statins and was not apparent in the larger cohort of those not prescribed statins. This argues against a relationship between the genotype and the outcome which is not drug mediated.

## Conclusions

Our study shows an association between statin use and increased risk of non-senile cataracts is due to confounders linked with both cardiovascular/metabolic and cataract pathophysiology, in keeping with previous research. We hereby demonstrate on a large cohort that the SLCO1B1*5 genotype, known to lead to increased statin exposure, is significantly associated with decreased risk of non-senile cataracts in those taking statins. Although our novel results will need to be validated in other cohorts, they emphasize a new approach to a controversial question, utilizing a well characterized pharmacogene, and can provide two important clinical points. The first is re-assurance to patients and cardiometabolic clinicians who take and prescribe statins regularly, that this study agrees with several prior studies in concluding that statin use is not associated independently with increased risk of cataracts. The second is support for a protective association between statins and cataracts for those at high risk of non-senile cataracts due to comorbidities and exposed to higher concentration of drug. Furthermore, stratification of on-drug cohorts by validated pharmacogenomic variants is a useful tool to support or repudiate adverse drug events in observational cohorts. The population level protective effect of SLCO1B1*5 in statin users, would be more pronounced in ethnic cohorts with higher prevalence of the *5 allele, such as European ancestry populations, assuming equal prescribing prevalence and morbidity burden.

## Supplementary information

Supplementary materials

## Data Availability

All Genes & Health data can be accessed by application to the study access team https://www.genesandhealth.org/research/scientists-using-genes-health-scientific-research.
