# Supplementary material for: SLCO1B1*5 is protective against non-senile cataracts in cohort prescribed statins: analysis in a British-South Asian cohort
Source: Pharmacogenomics J. 2023 May 23;23(5):134–9. doi: 10.1038/s41397-023-00307-w (PMC10506906; doi:10.1038/s41397-023-00307-w)
Supplement: Supplementary file 1 — Supplementary materials [file 41397_2023_307_MOESM1_ESM.docx]

**Supplementary tables**

**Supplementary table 1**- Characteristics of *5 Genotype

| Cohort | **HWE *5** | **Fractional Missingness *5** | **MAF *5** |
| --- | --- | --- | --- |
| **Ever-prescribed Statin Cohort** (N=102,704) | 1 | 0.0003 | 0.045 |
| **Never-prescribed Statin** (N=23,809) | 0.2 | 8e-5 | 0.043 |
| **Total Cohort** (N=36,513) | 0.32 | 0.0002 | 0.044 |

**Supplementary table 2 -** Cohort demographics stratified by genotype in:

Participants prescribed statins (N 12704) and participants not prescribed statins (N 23809)

|  | **Stratification of participants prescribed statin by genotype**  **(N** 12704) | | | **Stratification of participants not prescribed statin by genotype**  **(N** 23809) | | |
| --- | --- | --- | --- | --- | --- | --- |
| Genotype | *SLCO1B1*5* present (N 1115) | *SLCO1B1*5* absent (N 11589) | P value | *SLCO1B1*5* present (N 2007) | *SLCO1B1*5* absent (N 21802) | P value |
| Obesity % (n) | 21 (234) | 23 (2694) | 0.09 | 14 (273) | 13 (2939) | 0.9 |
| Diabetes % (n) | 39 (430) | 43 (4947) | 0.008 | 3 (59) | 3 (588) | 0.5 |
| HTN % (n) | 44 (491) | 46 (5370) | 0.15 | 4 (86) | 3 (854) | 0.4 |
| Dyslipidemia % (n) | 51 (570) | 54 (6261) | 0.06 | 3 (59) | 3 (686) | 0.7 |
| CKD % (n) | 15 (162) | 15 (1773) | 0.5 | 0.8 (16) | 0.9 (204) | 0.6 |
| PVD % (n) | 2 (22) | 2 (252) | 0.7 | 0.8 (16) | 0.9 (187) | 0.9 |
| IHD % (n) | 20 (224) | 21 (2405) | 0.6 | 0.7 (14) | 0.4 (98) | 0.1 |
| Cataracts (all) % (n) | 10 (115) | 14 (1649) | 0.0002 | 0.8 (17) | 0.9 (192) | 1 |
| Cataracts, non-senile % (n) | 8 (94) | 12 (1413) | 0.0002 | 0.6 (12) | 0.8 (167) | 0.5 |
| Male % (n) | 59 (656) | 58 (6739) | 0.68 | 40 (797) | 38 (8273) | 0.1 |
| Average age at enrollment | 53 years old | 53 years old | 0.06 | 34 years old | 34 years old | 0.7 |

Current Genes & Health Research Team (in alphabetical order by surname):   Shaheen Akhtar, Mohammad Anwar, Elena Arciero, Omar Asgar, Samina Ashraf, Saeed Bidi, Gerome Breen, Raymond Chung, David Collier, Charles J Curtis, Shabana Chaudhary, Megan Clinch, Grainne Colligan, Panos Deloukas, Ceri Durham, Faiza Durrani, Fabiola Eto, Sarah Finer, Joseph Gafton, Ana Angel Garcia, Chris Griffiths, Joanne Harvey, Teng Heng, Sam Hodgson, Qin Qin Huang, Matt Hurles, Karen A Hunt, Shapna Hussain, Kamrul Islam, Vivek Iyer, Ben Jacobs, Ahsan Khan, Cath Lavery, Sang Hyuck Lee, Robin Lerner, Daniel MacArthur, Daniel Malawsky, Hilary Martin, Dan Mason, Rohini Mathur, Mohammed Bodrul Mazid, John McDermott, Caroline Morton, Bill Newman, Elizabeth Owor, Asma Qureshi, Samiha Rahman, Shwetha Ramachandrappa, Mehru Reza, Jessry Russell, Nishat Safa, Miriam Samuel, Michael Simpson, John Solly, Marie Spreckley. Daniel Stow, Michael Taylor, Richard C Trembath, Karen Tricker, Nasir Uddin, David A van Heel, Klaudia Walter, Caroline Winckley, Suzanne Wood, John Wright, Julia Zollner.
